# Living at home after emergency hospital admission: prospective cohort study in older adults with and without cognitive spectrum disorder

**DOI:** 10.1186/s12916-018-1199-z

**Published:** 2018-12-11

**Authors:** Jennifer K. Burton, Bruce Guthrie, Simona M. Hapca, Vera Cvoro, Peter T. Donnan, Emma L. Reynish

**Affiliations:** 1 Academic Geriatric Medicine, Institute of Cardiovascular and Medical Sciences, University of Glasgow, New Lister Building Glasgow Royal Infirmary, 10 Alexandra Parade, G31 2ER Glasgow, Scotland; 2 Population Health Sciences Division, University of Dundee, Mackenzie Building, Kirsty Semple Way, Dundee, DD2 4BF Scotland; 3 NHS Fife, Kirkcaldy, Fife, KY2 5AH Scotland; 4 Dementia and Ageing Research Group, Faculty of Social Science, University of Stirling, Stirling, FK9 4LA Scotland

**Keywords:** Cognitive spectrum disorder, Dementia, Delirium, Delirium superimposed on dementia, Care home, Long-term care, Nursing home, Outcome, Data linkage

## Abstract

**Background:**

Cognitive spectrum disorders (CSDs) are common in hospitalised older adults and associated with adverse outcomes. Their association with the maintenance of independent living has not been established. The aim was to establish the role of CSDs on the likelihood of living at home 30 days after discharge or being newly admitted to a care home.

**Methods:**

A prospective cohort study with routine data linkage was conducted based on admissions data from the acute medical unit of a district general hospital in Scotland. 5570 people aged ≥ 65 years admitted from a private residence who survived to discharge and received the Older Persons Routine Acute Assessment (OPRAA) during an incident emergency medical admission were included.

The outcome measures were living at home, defined as a private residential address, 30 days after discharge and new care home admission at hospital discharge. Outcomes were ascertained through linkage to routine data sources.

**Results:**

Of the 5570 individuals admitted from a private residence who survived to discharge, those without a CSD were more likely to be living at home at 30 days than those with a CSD (93.4% versus 81.7%; difference 11.7%, 95%CI 9.7–13.8%). New discharge to a care home affected 236 (4.2%) of the cohort, 181 (76.7%) of whom had a CSD. Logistic regression modelling identified that all four CSD categories were associated with a reduced likelihood of living at home and an increased likelihood of discharge to a care home. Those with delirium superimposed on dementia were the least likely to be living at home (OR 0.25), followed by those with dementia (OR 0.43), then unspecified cognitive impairment (OR 0.55) and finally delirium (OR 0.57).

**Conclusions:**

Individuals with a CSD are at significantly increased risk of not returning home after hospitalisation, and those with CSDs account for the majority of new admissions to care homes on discharge. Individuals with delirium superimposed on dementia are the most affected. We need to understand how to configure and deliver healthcare services to enable older people to remain as independent as possible for as long as possible and to ensure transitions of care are managed supportively.

## Background

Older adults admitted on an unplanned basis occupy the majority of emergency hospital bed days in the UK [1]. Reasons for admission are broad and include a range of conditions requiring treatment, including infections, cardiovascular and cerebrovascular events, cancers, injuries and poisoning [2]. The aim of effective acute hospital care for older people is to assess individual needs, treat modifiable conditions, support functional recovery and facilitate discharge.

Cognitive spectrum disorders (CSD) is a term encompassing diagnosed dementia, delirium, delirium superimposed on known dementia and unspecified cognitive impairment [3]. CSDs are common in hospitalised older adults, affecting 38.5% of over 65 year olds with an emergency medical admission [3]. Despite high prevalence, cognitive impairment is often unrecognised in the acute hospital setting [4]. Those whose cognitive function worsens in hospital are at particular risk of functional decline as an inpatient [5]. Mortality during and after admission is higher in people with cognitive impairment than those without, and is increased irrespective of the cause of cognitive impairment [6], with mortality 12 months after discharge in our cohort of 40% in older adults with CSD compared to 26% for those without [3].

National health policy promotes that health services should enable older people to remain as independent as possible [7, 8], and a key outcome after hospital admission is the maintenance of independence. The preference for care at home is shared among older adults and family carers [9, 10]. Returning to and remaining at home after acute hospital care is therefore a highly desirable outcome for patients, health services and society.

New care home admission can be necessary to address care needs which cannot be met in the community but is a significant and life-changing event which many older people fear. Care home admission from hospital is common although rates vary significantly between hospitals [11, 12] and may happen prematurely in people with dementia [13]. As well as age and functional impairment, dementia is an established predictor of new care home admission, but it is less clear that the extent to which other forms of cognitive impairment are associated with care home admission [14].

## Methods

### Aim

The aim of this study was to use data from a large population cohort of older people with an emergency hospital admission to examine associations between CSDs and the two outcomes of living at home 30 days after discharge and new care home admission at the time of hospital discharge.

### Design, participants and setting

The overall design is a prospective cohort study of people aged ≥ 65 years to the Acute Medical Unit (AMU) of a district general hospital in Kirkcaldy, East of Scotland. The hospital is the sole provider of emergency medical care for a local population of ~ 370,000 [15], and almost all such admissions start in AMU. From 1 January 2012 to 31 December 2013, incident admissions of adults aged ≥ 65 years were included if patients were admitted from their own home, received an Older Persons Routine Acute Assessment (OPRAA) during admission, and survived to be discharged. Incident admissions were defined as the first admission to the AMU in the study period where there had not been a previous AMU admission in the preceding 6 months. OPRAA assessment was based on the principles of Comprehensive Geriatric Assessment [16], and was performed by Specialist Nurses trained in the use of the measures described below. By design, OPRAA was *not* carried out in those with a predicted admission of < 24 h, those in whom death was considered imminent and those requiring admission to critical care.

OPRAA assessment data were linked to several routine datasets: the Scottish Morbidity Record SMR01 and SMR04 which records all hospital admissions and day-case attendances for medicine/surgery and psychiatry respectively; community-dispensed prescribing data; and the Community Health Index (CHI-the NHS Scotland patient register). This linkage provided information on all hospital activity before and after the acute admission and allowed ascertainment of mortality and care home residency status.

### Outcome assessment

The primary outcome was living at home 30 days after discharge. Living at home was defined as not living in an institutional care setting. The secondary outcome was new care home admission at the point of hospital discharge. These outcomes required allocation of residency status at admission and discharge; calculation and exclusion of in-hospital mortality and evaluation of mortality and residential status at 30 days after discharge. Care home residency was established by identifying addresses recorded in the CHI register as any nursing or residential care facility providing 24-h care for its residents. Mortality data were obtained from the CHI register and re-admissions were ascertained using SMR01 data.

### Other variables

Demographic information was extracted from the CHI register. Deprivation was measured using the Scottish Index of Multiple Deprivation (SIMD), an area-based measure of deprivation categorised into five equal groups (quintiles) [17]. The Charlson Comorbidity Index [18] was calculated using ICD-10 codes from SMR01. Scoring was adjusted to remove dementia as this was evaluated separately. The number of medications was calculated using community prescribing data and this was categorised based on the number of dispensed items in the preceding 12 weeks before admission. Cognitive and functional status were defined as reported in Table 1.

**Table 1:** Definitions of cognitive and functional status used in the OPRAA cohort

Cognitive status classification	
•Known dementia: diagnosis of dementia recorded in SMR01 or SMR04, or prescription for cognitive enhancing medications (anticholinesterase inhibitor or memantine) in community prescribing data, or self or informant report of diagnosed dementia in OPRAA	
•Delirium: presence of full syndromic delirium based on positive score using the Confusion Assessment Method [39], or a clinical diagnosis of delirium made by specialist nurse assessment	
•Delirium superimposed on known dementia: combination of first two categories	
•Unspecified cognitive impairment: abbreviated mental test score [40] of <8/10 in the absence of a diagnosis of dementia or delirium	
Functional status classification	
Activities of daily living (ADL) status was assessed using the Katz Index which assesses independence in six domains, with a maximum of six points for independence in all domains [41]. Evaluation was made about the Katz Index on admission and 3 months prior to admission, based on patient or informant response. Three categories were created:	
•Persistently high ADL score ≥ 5 both prior to and on admission	
•Acutely changed ADL score ≥ 5 3 months before admission with score < 5 on admission	
•Persistently low ADL score < 5 both prior to and on admission	

### Patient and public involvement (PPI)

PPI representatives were involved in the External Advisory Board of the project and one former carer joined the Research Team, attending project meetings and contributing to the interpretation of findings.

### Permissions

The University of Dundee Health Informatics Centre (HIC) performed the data linkage and provided an anonymised dataset in a secure Scottish Government accredited safe haven environment (HIC ISO27001) for analysis by the research team. HIC Standard Operating Procedures have been reviewed and approved by NHS East of Scotland Research Ethics Service. Permission for this project was granted by the NHS Fife Caldicott Guardian.

### Statistical methods

Descriptive statistics were used to compare the distribution of baseline characteristics in those with and without a CSD. Mean age and standard deviation are reported for each group. The primary and secondary outcomes are binary and analysis therefore used logistic regression to examine unadjusted and adjusted associations with patient characteristics including the presence of a CSD. Logistic regression models were reported as odds ratios (ORs) and their associated 95% confidence intervals (95%CI). All baseline characteristic variables were included in the adjusted analyses including age categories (65–74; 75–84 and ≥ 85 years); sex; area-based deprivation quintiles; Charlson Comorbidity Index categories (0; 1; 2–5 and ≥ 6); community prescribing medication count categories (0–5; 6–10 and > 10) and activities of daily living (ADL) categories (see Table 1).

All analysis was performed in SAS® version 9.4. Multiple imputation was used to impute the 28.6% of missing values for ADL 3 months before and at admission. A sensitivity analysis was also performed that used complete case analysis.

## Results

During the study period, there were 9331 incident admissions of whom 6724 (72.1%) received an OPRAA. From this group, 500 were admitted from a care home and 654 died during the admission. In total, 5570 individuals admitted from a private residence, surviving to discharge and with an OPRAA were included in analysis. Included participants were majority women (56.3%), with Charlson Index scores of two or more (79.7%) and prescription of more than six medications before hospitalisation (68.7%). Those with CSD had a mean age of 81.7 years [SD 7.8] compared to those without a CSD 77.8 years [SD 7.6]. CSD was present in 29.9% (95%CI 28.8–31.1). Of those with CSD, 49.3% (95%CI 46.9–51.7) had delirium alone, 20.0% (95%CI 18.3–22.1) had known dementia, 17.1% (95%CI 15.4–19.0) had delirium superimposed on known dementia and 13.6% (95%CI 12.0–15.3) had unspecified cognitive impairment (Table 2).

**Table 2:** Summary of characteristics of included population (incident admissions admitted from private residence and discharged alive) (*n* = 5570)

	No cognitive spectrum disorders	Any cognitive spectrum disorders	Cognitive spectrum disorders			
			Delirium alone	Known dementia alone	Delirium superimposed on known dementia	Unspecified cognitive impairment
	No. (%)	No. (%)	No. (%)	No. (%)	No. (%)	No. (%)
	*N* = 3903	*N* = 1667	*N* = 821	*N* = 335	*N* = 285	*N* = 226
	(70.1%; 95%CI 68.9–71.3 of all patients)	(29.9%; 95%CI 28.8–31.1 of all patients)	(14.7%; 95%CI 13.8–15.7 of all patients)	(6.0%; 95%CI 5.4–6.7 of all patients)	(5.1%; 95%CI 4.6–5.7 of all patients)	(4.1%; 95%CI 3.6–4.6 of all patients)
Age						
65–74 (*n* = 1777)	1454 (37.3)	323 (19.4)	207 (25.2)	47 (14.0)	31 (10.9)	38 (16.8)
75–84 (*n* = 2318)	1635 (41.9)	683 (41.0)	323 (39.3)	146 (43.6)	122 (42.8)	92 (40.7)
≥ 85 (*n* = 1475)	814 (20.9)	661 (39.7)	291 (35.4)	142 (42.3)	132 (46.3)	96 (42.5)
Sex						
Women (*n* = 3138)	2143 (54.9)	995 (59.6)	473 (57.6)	207 (61.8)	182 (63.9)	133 (58.8)
Men (*n* = 2432)	1760 (45.1)	672 (40.3)	348 (42.4)	128 (38.2)	103 (36.1)	93 (41.2)
Deprivation SIMD quintile^a^						
1 most deprived (*n* = 1151)	824 (21.1)	327 (19.6)	179 (21.8)	60 (17.9)	44 (15.4)	44 (19.5)
2 (*n* = 1473)	1013 (26.0)	460 (27.6)	233 (28.4)	81 (24.2)	71 (24.9)	75 (33.2)
3 (*n* = 1266)	884 (22.6)	382 (22.9)	178 (21.7)	94 (28.1)	61 (21.4)	49 (21.7)
4 (*n* = 853)	579 (14.8)	274 (16.4)	129 (15.7)	51 (15.2)	66 (23.2)	28 (12.4)
5 most affluent (*n* = 827)	603 (15.5)	224 (13.4)	102 (12.4)	49 (14.6)	43 (15.1)	30 (13.3)
Charlson Comorbidity Index (CCI)^b^						
CCI 0 (*n* = 1424)	950 (24.3)	474 (28.4)	210 (25.6)	113 (33.7)	101 (35.4)	50 (22.1)
CCI 1 (*n* = 1496)	1082 (27.7)	414 (24.8)	209 (25.5)	85 (25.4)	56 (19.6)	64 (28.3)
CCI 2 to 5 (*n* = 2233)	1548 (39.7)	685 (41.1)	350 (42.6)	124 (37.0)	118 (41.4)	93 (41.2)
CCI ≥6 (*n* = 417)	323 (8.3)	94 (5.6)	52 (6.3)	13 (3.9)	10 (3.5)	19 (8.4)
No. of medications						
0–5 (*n* = 1746)	1216 (31.2)	530 (31.8)	244 (29.7)	107 (31.9)	108(37.9)	71 (31.4)
6–10 (*n* = 2204)	1552 (39.8)	652 (39.1)	334 (40.7)	134 (40.0)	105 (36.8)	79 (35.0)
> 10 (*n* = 1620)	1135 (29.1)	485 (29.1)	243 (29.6)	94 (28.1)	72 (25.3)	76 (33.6)
Activities of daily living (ADL)^c^						
Persistently high ADL (*n* = 1956)	1611 (62.3)	345 (24.7)	155 (24.7)	77 (28.5)	44 (15.9)	69 (31.4)
Changed ADL (*n* = 1341)	742 (28.7)	599 (43.0)	322 (51.4)	76 (28.1)	107 (38.6)	94 (42.7)
Persistently low ADL (*n* = 681)	231 (8.9)	450 (32.3)	150 (23.9)	117 (43.3)	126 (45.5)	57 (25.9)
Discharge destination						
Private home (*n* = 5334)	3848 (98.6)	1486 (89.1)	769 (93.7)	299 (89.3)	210 (73.7)	208 (92.0)
New care home admission (*n* = 236)	55 (1.4)	181 (10.9)	52 (6.3)	36 (10.7)	75 (26.3)	18 (8.0)

^a^SIMD quintile: Scottish Index of Multiple Deprivation

^b^Charlson Comorbidity Index groups based on ICD10 coding in SMR01 data, omitting dementia

^c^Activities of daily living classification based on Katz Index score on admission and 3 months prior to admission; data available for 3978 (71%)

### Primary outcome—living at home at 30 days after discharge

**Fig. 1:**
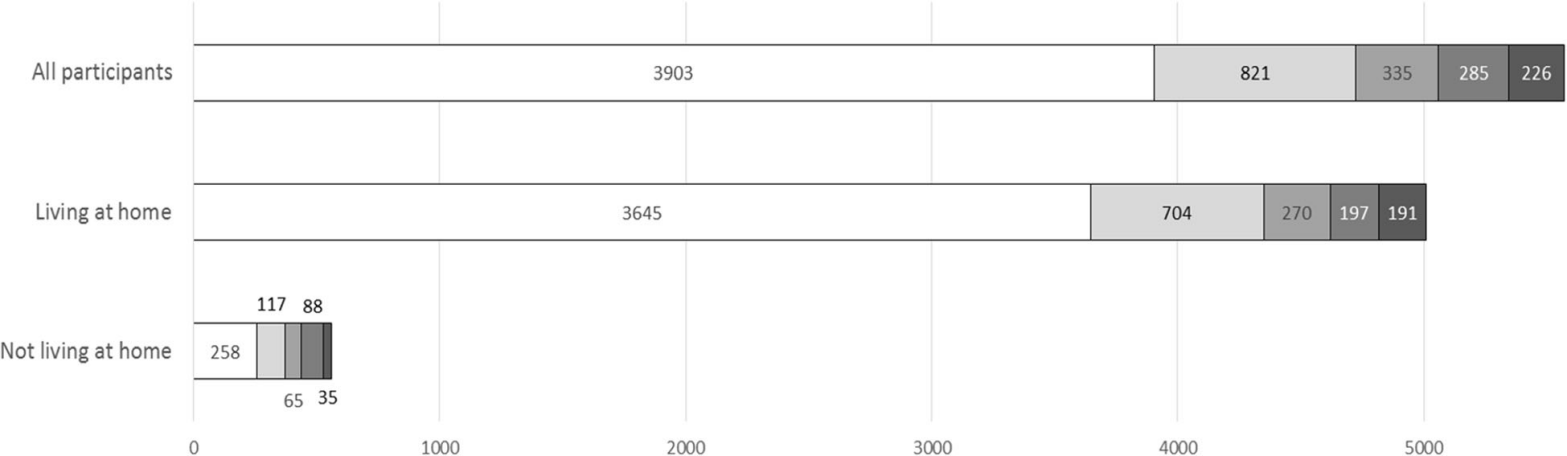
Distribution of primary outcome by cognitive spectrum disorder

5007 (89.9%, 95%CI 89.1–90.6) patients were living at home 30 days after discharge. Of the 563 not living at home, 122 had died in the 30 days following discharge, 213 were hospital in-patients and 228 were care home residents. Patients without a CSD were more likely to be living at home than those with a CSD (93.4% vs 81.7%, difference 11.7%, 95%CI 9.7–13.8%). Table 3 and Fig. 1 show the distribution of outcomes by CSD. Of those with CSD, those with delirium (85.8%, 95%CI 83.2–88.0) or unspecified cognitive impairment (84.5%, 95%CI 79.2–88.7) were the most likely to be living at home, with lower proportions for dementia alone (80.6%, 95%CI 76.0–84.4) and particularly delirium superimposed on dementia (69.1%, 95%CI 63.5–74.2).

**Table 3:** Distribution of outcomes at 30 days after discharge by cognitive spectrum disorder

	No cognitive spectrum disorders	Any cognitive spectrum disorders	Delirium alone	Known dementia alone	Delirium superimposed on known dementia	Unspecified cognitive impairment
	No. of patients (%; 95%CI)	No. of patients (%; 95%CI)	No. of patients (%; 95%CI)	No. of patients (%; 95%CI)	No. of patients (%; 95%CI)	No. of patients (%; 95%CI)
Living at home (*n* = 5007)	3645 (93.4; 92.6–94.1)	1362 (81.7; 79.8–83.5)	704 (85.8; 83.2–88.0)	270 (80.6; 76.0–84.4)	197 (69.1; 63.5–74.2)	191 (84.5; 79.2–88.7)
Not living at home						
All (*n* = 563)	258 (6.6; 5.9–7.4)	305 (18.3; 16.5–20.2)	117 (14.3; 12.0–16.8)	65 (19.4; 15.5–24.0)	88 (30.9; 25.8–36.5)	35 (15.5; 11.4–20.8)
Dead (*n* = 122)	85 (2.2; 1.8–2.7)	37 (2.2; 1.6–3.0)	20 (2.4; 1.6–3.7)	7 (2.1; 1.0–4.3)	4 (1.4; 0.6–3.6)	6 (2.7; 1.2–5.7)
In-hospital (*n* = 213)	120 (3.1; 2.6–3.7)	93 (5.6; 4.6–6.8)	48 (5.9; 4.4–7.7)	23 (6.9; 4.6–10.1)	11 (3.9; 2.2–6.8)	11 (4.9; 2.7–8.5)
Care home (*n* = 228)	53 (1.4; 1.0–1.8)	175 (10.5; 9.1–12.1)	49 (6.0; 4.6–7.8)	35 (10.5; 7.6–14.2)	73 (25.6; 20.9–31.0)	18 (8.0; 5.1–12.2)

*95%CI* 95% confidence interval

Univariate analysis identified increased age, Charlson Index score ≥ 6, all four CSD categories and persistently low or acutely changed ADL scores as being associated with a reduced likelihood of the positive outcome of living at home 30 days after discharge. These factors remained statistically significantly associated after adjustment. All four CSD categories were associated with a lower chance of experiencing a positive outcome. Those with delirium superimposed on dementia were the least likely to experience a positive outcome (OR 0.25, 95%CI 0.18–0.34), followed by those with dementia alone (OR 0.43 95%CI 0.31–0.59), then those with unspecified cognitive impairment (OR 0.55 95%CI 0.37–0.82) and those with delirium alone (OR 0.57 95%CI 0.44–0.72) after adjustment for all other variables.

Older adults aged 74–84 (OR 0.64 95%CI 0.49–0.83) and ≥ 85 years (OR 0.43 95%CI 0.33–0.56), those with a Charlson Index score of ≥ 6 (OR 0.34 95%CI 0.24–0.47), those whose ADL scores were persistently low (OR 0.63 95%CI 0.51–0.78) or whose ADL scores acutely changed (OR 0.41 95%CI 0.32–0.52) were less likely to experience a positive outcome (Table 4).

**Table 4:** Associations between patient characteristics and a positive outcome (living at home versus in hospital, newly admitted to a care home or dying after discharge) at 30 days after discharge (*n* = 5007)

Variables	N (%; 95%CI) with positive outcome at 30 days	Unadjusted model	Adjusted model
	Total *N* = 5007	Odds ratio (95% confidence interval)	
Age			
65–74 (*n* = 1777)	1683 (94.7; 93.6–95.7)	1	1
75–85 (*n* = 2318)	2092 (90.3; 89.0–91.4)	*0.52 (0.40–0.66)*	*0.64 (0.49–0.83)*
≥ 85 (*n* = 1475)	1232 (83.5; 81.6–85.3)	*0.28 (0.22–0.36)*	*0.43 (0.33–0.56)*
Sex			
Women (*n* = 3138)	2811 (89.6; 88.5–90.6)	1	1
Men (*n* = 2432)	2196 (90.3; 89.0–91.4)	1.08 (0.91–1.29)	0.90 (0.75–1.09)
Deprivation (SIMD quintile)			
1 most deprived (*n* = 1151)	1054 (91.6; 89.8–93.0)	1.16 (0.85–1.59)	1.11 (0.81–1.54)
2 (*n* = 1473)	1323 (89.8; 88.2–91.3)	0.95 (0.71–1.26)	0.92 (0.69–1.25)
3 (*n* = 1266)	1131 (89.3; 87.5–90.9)	0.90 (0.67–1.20)	0.92 (0.68–1.25)
4 (*n* = 853)	752 (88.2; 85.8–90.2)	0.80 (0.58–1.09)	0.88 (0.64–1.22)
5 most affluent (*n* = 827)	747 (90.3; 88.1–92.2)	1	1
Charlson Comorbidity Index (CCI)			
CCI 0 (*n* = 1424)	1284 (90.2; 88.5–91.6)	1	1
CCI 1 (*n* = 1496)	1357 (90.7; 89.1–92.1)	1.06 (0.83–1.36)	0.87 (0.67–1.13)
CCI 2 to 5 (*n* = 2233)	2024 (90.6; 89.4–91.8)	1.06 (0.84–1.32)	0.97 (0.76–1.23)
CCI ≥ 6 (*n* = 417)	342 (82.0; 78.0–85.4)	*0.50 (0.37–0.67)*	*0.34 (0.24–0.47)*
Cognitive spectrum disorder			
None (*n* = 3903)	3645 (93.4; 92.6–94.1)	1	1
Delirium alone (*n* = 821)	704 (85.7; 83.2–88.0)	*0.43 (0.34–0.54)*	*0.57 (0.44–0.72)*
Known dementia (*n* = 335)	270 (80.6; 76.0–84.5)	*0.29 (0.22–0.40)*	*0.43 (0.31–0.59)*
Delirium superimposed on dementia (*n* = 285)	197 (69.1; 63.4–74.2)	*0.16 (0.12–0.21)*	*0.25 (0.18–0.34)*
Unspecified cognitive impairment (*n* = 226)	191 (84.5; 79.2–88.7)	*0.39 (0.26–0.57)*	*0.55 (0.37–0.82)*
No. of medications			
0–5 (*n* = 1746)	1571 (90.0; 88.5–91.3)	1	1
6–10 (*n* = 2204)	1965 (89.2; 87.8–90.4)	0.92 (0.75–1.13)	0.96 (0.77–1.19)
> 10 (*n* = 1620)	1471 (90.8;89.3–92.1)	1.10 (0.87–1.38)	1.15 (0.90–1.48)
Activities of daily living (ADL) score			
Persistently high ADL (*n* = 1956)	1835 (93.8; 92.6–94.8)	1	1
Changed ADL (*n* = 1341)	1156 (86.2; 84.3–87.9)	*0.20 (0.16–0.26)*	*0.41 (0.32–0.52)*
Persistently low ADL (*n* = 681)	515 (75.6; 72.2–78.8)	*0.41 (0.30–0.52)*	*0.63 (0.51–0.78)*

Italic text denotes results which are statistically significant

*95%CI* 95% confidence interval; *SIMD* Scottish Index of Multiple Deprivation

### New care home admission at hospital discharge

236 (4.2%, 95%CI 3.7–4.8%) patients were newly admitted to a care home on discharge from hospital. Univariate analysis identified that CSDs, increased age and acutely changed ADL score or persistently low ADL scores were all associated with an increased likelihood of new care home admission and that being male, living in the most deprived areas, having comorbidities and being prescribed > 10 medications were associated with lower risk. Associations with comorbidity were not statistically significant after adjustment for other variables.

All CSDs were associated with an increased likelihood of care home admission at the time of discharge. Those with delirium superimposed on dementia were at the greatest risk (OR 11.72 95%CI 7.82–17.56), followed by those with dementia alone (OR 4.28 95%CI 2.69–6.82), then those with unspecified cognitive impairment (OR 3.65 95%CI 2.06–6.47) and those with delirium alone (OR 3.04 95%CI 2.03–2.47). Older adults aged 75–84 (OR 3.79 95%CI 2.04–7.07) ≥ 85 years (OR 6.43 95%CI 3.47–11.89) and those whose ADL scores were persistently low (OR 1.89 95%CI 1.31–2.71) or acutely changed (OR 2.99 95%CI 2.06–4.36) were more likely to be admitted to a care home at the time of discharge (Table 4). Men, individuals living in the most deprived areas and those prescribed > 10 medications before admission were less likely to be newly admitted to a care home on hospital discharge (Table 5).

**Table 5:** Predictors of new care home admission at time of discharge (*n* = 236)

Variables	*N* (%; 95%CI) with new care home admission at discharge	Unadjusted model	Adjusted model
	Total *N* = 236	Odds ratio (95% confidence interval)	
Age			
65–74 (*n* = 1777)	12 (0.7; 0.4–1.2)	1	1
75–84 (*n* = 2318)	84 (3.6; 2.9–4.5)	*5.53 (3.01–10.16)*	*3.79 (2.04–7.07)*
≥ 85 (*n* = 1475)	140 (9.5; 8.1–11.1)	*15.43 (8.52–27.93)*	*6.43 (3.47–11.89)*
Sex			
Women (*n* = 3138)	167 (5.3; 4.6–6.2)	1	1
Men (*n* = 2432)	69 (2.8; 2.3–3.6)	*0.52 (0.39–0.69)*	*0.68 (0.50–0.93)*
Deprivation (SIMD quintile)			
1 most deprived (*n* = 1151)	26 (2.3; 1.6–3.3)	*0.49 (0.30–0.82)*	*0.52 (0.30–0.90)*
2 (*n* = 1473)	56 (3.8; 2.9–4.9)	0.84 (0.55–1.29)	0.88 (0.56–1.39)
3 (*n* = 1266)	62 (4.9; 3.8–6.2)	1.10 (0.73–1.67)	1.02 (0.65–1.61)
4 (*n* = 853)	55 (6.4; 5.0–8.3)	1.47 (0.96–2.26)	1.26 (0.79–2.01)
5 most affluent (*n* = 827)	37 (4.5; 3.3–6.1)	1	1
Charlson Comorbidity Index (CCI)			
CCI 0 (*n* = 1424)	83 (5.8; 4.7–7.2)	1	1
CCI 1 (*n* = 1496)	58 (3.9; 3.0–5.0)	*0.65 (0.46–0.92)*	1.00 (0.68–1.46)
CCI 2 to 5 (*n* = 2233)	83 (3.7; 3.0–4.6)	*0.62 (0.46–0.85)*	0.87 (0.61–1.23)
CCI ≥ 6 (*n* = 417)	12 (2.9; 1.7–5.0)	*0.48 (0.26–0.89)*	0.98 (0.50–1.90)
Cognitive spectrum disorder			
None (*n* = 3903)	55 (1.4; 1.1–1.8)	1	1
Delirium alone (*n* = 821)	52 (6.3; 4.9–8.2)	*4.73 (3.21–6.97)*	*3.04 (2.03–4.57)*
Known dementia (*n* = 335)	36 (10.7; 7.9–14.5)	*8.43 (5.45–13.03)*	*4.28 (2.69–6.82)*
Delirium superimposed on dementia (*n* = 285)	75 (26.3; 21.6–31.7)	*25.00 (17.18–36.34)*	*11.72 (7.82–17.56)*
Unspecified cognitive impairment (*n* = 226)	18 (8.0; 5.1–12.2)	*6.05 (3.49–10.50)*	*3.65 (2.06–6.47)*
No. of medications			
0–5 (*n* = 1746)	99 (5.7; 4.7–6.9)	1	1
6–10 (*n* = 2204)	97 (4.4; 3.6–5.3)	0.77 (0.57–1.02)	0.74 (0.53–1.02)
> 10 (*n* = 1620)	40 (2.5; 1.8–3.4)	*0.42 (0.29–0.61)*	*0.42 (0.28–0.64)*
Activities of daily living (ADL) score			
Persistently high ADL (*n* = 1956)	31 (1.6; 1.1–2.2)	1	1
Changed ADL (*n* = 1341)	92 (6.9; 5.6–8.3)	*4.55 (3.45–6.00)*	*2.99 (2.06–4.36)*
Persistently low ADL (*n* = 681)	102 (15.0; 12.5–17.9)	*1.39 (1.05–1.82)*	*1.89 (1.31–2.71)*

Italic text denotes results which are statistically significant

*95%CI* 95% confidence interval; *SIMD* Scottish Index of Multiple Deprivation

### Sensitivity analysis

Complete case analysis excluding those with missing ADL data found consistent results with the main analysis (Tables 6 and 7).

**Table 6:** Sensitivity analysis of complete case analysis compared to imputed ADL adjusted model with respect to primary outcome (*n* = 5007)

Variables	*N* (%; 95%CI) with positive outcome at 30 days	Adjusted model	Sensitivity analysis* Complete case analysis
	Total *N* = 5007	Odds ratio (95% confidence interval)	
Age			
65–74 (*n* = 1777)	1683 (94.7; 93.6–95.7)	1	1
75–85 (*n* = 2318)	2092 (90.3; 89.0–91.4)	*0.64 (0.49–0.83)*	*0.68 (0.51–0.92)*
≥ 85 (*n* = 1475)	1232 (83.5; 81.6–85.3)	*0.43 (0.33–0.56)*	*0.48 (0.35–0.65)*
Sex			
Women (*n* = 3138)	2811 (89.6; 88.5–90.6)	1	1
Men (*n* = 2432)	2196 (90.3; 89.0–91.4)	0.90 (0.75–1.09)	0.91 (0.74–1.13)
Deprivation (SIMD quintile)			
1 most deprived (*n* = 1151)	1054 (91.6; 89.8–93.0)	1.11 (0.81–1.54)	1.32 (0.92–1.89)
2 (*n* = 1473)	1323 (89.8; 88.2–91.3)	0.92 (0.69–1.25)	0.91 (0.66–1.27)
3 (*n* = 1266)	1131 (89.3; 87.5–90.9)	0.92 (0.68–1.25)	1.04 (0.74–1.46)
4 (*n* = 853)	752 (88.2; 85.8–90.2)	0.88 (0.64–1.22)	0.93 (0.65–1.33)
5 most affluent (*n* = 827)	747 (90.3; 88.1–92.2)	1	1
Charlson Comorbidity Index (CCI)			
CCI 0 (*n* = 1424)	1284 (90.2; 88.5–91.6)	1	1
CCI 1 (*n* = 1496)	1357 (90.7; 89.1–92.1)	0.87 (0.67–1.13)	0.92 (0.69–1.23)
CCI 2 to 5 (*n* = 2233)	2024 (90.6; 89.4–91.8)	0.97 (0.76–1.23)	0.96 (0.74–1.26)
CCI ≥ 6 (*n* = 417)	342 (82.0; 78.0–85.4)	*0.34 (0.24–0.47)*	*0.35 (0.24–0.51)*
Cognitive spectrum disorder			
None (*n* = 3903)	3645 (93.4; 92.6–94.1)	1	1
Delirium alone (*n* = 821)	704 (85.7; 83.2–88.0)	*0.57 (0.44–0.72)*	*0.53 (0.41–0.70)*
Known dementia (*n* = 335)	270 (80.6; 76.0–84.5)	*0.43 (0.31–0.59)*	*0.46 (0.32–0.66)*
Delirium superimposed on dementia (*n* = 285)	197 (69.1; 63.4–74.2)	*0.25 (0.18–0.34)*	*0.29 (0.21–0.39)*
Unspecified cognitive impairment (*n* = 226)	191 (84.5; 79.2–88.7)	*0.55 (0.37–0.82)*	*0.60 (0.40–0.91)*
No. of medications			
0–5 (*n* = 1746)	1571 (90.0; 88.5–91.3)	1	1
6–10 (*n* = 2204)	1965 (89.2; 87.8–90.4)	0.96 (0.77–1.19)	1.08 (0.85–1.37)
> 10 (*n* = 1620)	1471 (90.8;89.3–92.1)	1.15 (0.90–1.48)	*1.42 (1.07–1.88)*
Activities of daily living (ADL) score			
Persistently high ADL (*n* = 1956)	1835 (93.8; 92.6–94.8)	1	1
Changed ADL (*n* = 1341)	1156 (86.2; 84.3–87.9)	*0.41 (0.32–0.52)*	*0.35 (0.26–0.46)*
Persistently low ADL (*n* = 681)	515 (75.6; 72.2–78.8)	*0.63 (0.51–0.78)*	*0.55 (0.43–0.71)*

Italic text denotes results which are statistically significant

*95%CI* 95% confidence interval; *SIMD* Scottish Index of Multiple Deprivation

*Complete case analysis based on data from *n* = 3978 (71.4%) who had complete data recorded

**Table 7:** Sensitivity analysis of complete case analysis compared to imputed ADL adjusted model with respect to care home admission at discharge (*n* = 236)

Variables	N (%; 95%CI) with new care home admission at discharge	Adjusted model	Sensitivity analysis* Complete case analysis
	Total *N* = 236	Odds ratio (95% confidence interval)	
Age			
65–74 (*n* = 1777)	12 (0.7; 0.4–1.2)	1	1
75–84 (*n* = 2318)	84 (3.6; 2.9–4.5)	*3.79 (2.04–7.07)*	*3.17 (1.69–5.96)*
≥ 85 (*n* = 1475)	140 (9.5; 8.1–11.1)	*6.43 (3.47–11.89)*	*4.54 (2.44–8.44)*
Sex			
Women (*n* = 3138)	167 (5.3; 4.6–6.2)	1	1
Men (*n* = 2432)	69 (2.8; 2.3–3.6)	*0.68 (0.50–0.93)*	0.73 (0.53–1.00)
Deprivation (SIMD quintile)			
1 most deprived (*n* = 1151)	26 (2.3; 1.6–3.3)	*0.52 (0.30–0.90)*	*0.50 (0.28–0.86)*
2 (*n* = 1473)	56 (3.8; 2.9–4.9)	0.88 (0.56–1.39)	0.86 (0.54–1.38)
3 (*n* = 1266)	62 (4.9; 3.8–6.2)	1.02 (0.65–1.61)	0.95 (0.60–1.52)
4 (*n* = 853)	55 (6.4; 5.0–8.3)	1.26 (0.79–2.01)	1.17 (0.72–1.89)
5 most affluent (*n* = 827)	37 (4.5; 3.3–6.1)	1	1
Charlson Comorbidity Index (CCI)			
CCI 0 (*n* = 1424)	83 (5.8; 4.7–7.2)	1	1
CCI 1 (*n* = 1496)	58 (3.9; 3.0–5.0)	1.00 (0.68–1.46)	0.97 (0.66–1.45)
CCI 2 to 5 (*n* = 2233)	83 (3.7; 3.0–4.6)	0.87 (0.61–1.23)	0.89 (0.62–1.28)
CCI ≥ 6 (*n* = 417)	12 (2.9; 1.7–5.0)	0.98 (0.50–1.90)	0.96 (0.48–1.92)
Cognitive spectrum disorder			
None (*n* = 3903)	55 (1.4; 1.1–1.8)	1	1
Delirium alone (*n* = 821)	52 (6.3; 4.9–8.2)	*3.04 (2.03–4.57)*	*2.93 (1.91–4.49)*
Known dementia (*n* = 335)	36 (10.7; 7.9–14.5)	*4.28 (2.69–6.82)*	*4.09 (2.51–6.67)*
Delirium superimposed on dementia (*n* = 285)	75 (26.3; 21.6–31.7)	*11.72 (7.82–17.56)*	*9.14 (5.98–13.99)*
Unspecified cognitive impairment (*n* = 226)	18 (8.0; 5.1–12.2)	*3.65 (2.06–6.47)*	*2.98 (1.66–5.35)*
No. of medications			
0–5 (*n* = 1746)	99 (5.7; 4.7–6.9)	1	1
6–10 (*n* = 2204)	97 (4.4; 3.6–5.3)	0.74 (0.53–1.02)	*0.70 (0.51–0.98)*
> 10 (*n* = 1620)	40 (2.5; 1.8–3.4)	*0.42 (0.28–0.64)*	*0.35 (0.23–0.54)*
Activities of daily living (ADL) score			
Persistently high ADL (*n* = 1956)	31 (1.6; 1.1–2.2)	1	1
Changed ADL (*n* = 1341)	92 (6.9; 5.6–8.3)	*2.99 (2.06–4.36)*	*4.12 (2.61–6.49)*
Persistently low ADL (*n* = 681)	102 (15.0; 12.5–17.9)	*1.89 (1.31–2.71)*	*2.51 (1.62–3.89)*

Italic text denotes results which are statistically significant

*95%CI* 95% confidence interval; *SIMD* Scottish Index of Multiple Deprivation

*Complete case analysis based on data from *n* = 3978 (71.4%) who had complete data record

## Discussion

### Statement of principal findings

Nine out of 10 older adults discharged alive achieved the positive outcome of living at home 30 days after discharge. Although all CSDs were associated with a reduced likelihood of a positive outcome, people with dementia alone and particularly those with delirium superimposed on dementia had the greatest risk of not living at home. People aged 65–74 versus ≥ 75 without a CSD whose hospitalisation was not associated with acute decline in ADL were more likely to achieve a positive outcome. No statistically significant associations were observed with sex, area-based deprivation and polypharmacy.

New care home admission was relatively uncommon (4.2%) but is an important outcome after acute hospitalisation. The CSDs were all associated with new care home admission, with those with delirium superimposed on dementia the most likely to be admitted to a care home on discharge. In addition, increasing age, acutely worsened or consistently poor performance of ADL were associated with new care home admission. Being male, living in an area of high material deprivation and polypharmacy were associated with a reduced likelihood of care home admission. No statistically significant associations were observed with the Charlson Index score.

### Strengths and weaknesses of the study

The study examines a large cohort of routine users of NHS hospital services rather than a more selected consented cohort, typically included in research studies. The assessments performed are consistent with routine use of the evidence-based approach of comprehensive geriatric assessment [16]. Outcome ascertainment used population register data to robustly evaluate mortality and residency status.

Weaknesses include that only 72.1% of older people admitted received an OPRAA assessment. By design, OPRAA was not intended to be delivered to all patients (excluding those with predicted length of stay < 24 h and admitted to coronary or intensive care), and ascertainment is as complete as the best research studies [19, 20]. Those whose admission is very short are likely to return to their previous place of residence. However, those surviving their admission to critical care may develop significant physical dependency and be more likely to require admission to a care home [21].

ADL data were missing for 28.6% of the cohort. Ascertainment of this variable requires an assessment of ADL 3 months before admission and so relies either on carer/family report or a coherent history from the patient, which are not always available in a very busy clinical environment. In previous research, up to a quarter of those with cognitive impairment do not have an available informant [22]. Multiple imputation was used to address missing data, and sensitivity analysis using complete cases had findings consistent with the imputed analysis. Finally, the low incidence of new care home admission within the cohort results in wide confidence intervals for odds ratio estimates. Further research is needed in larger cohorts, but the associations identified are clinically plausible and identify a variation in risk depending on cognitive diagnosis and functional status.

More recently, attention has been focused on the association between frailty and adverse outcomes after hospitalisation [23] and among community-dwelling older adults [24]. Both these studies demonstrated practical measures to categorise frailty status, which can therefore be measured and adjusted-for in statistical modelling. No routine measure for categorising frailty was in operation during the study period and thus this variable cannot be formally quantified in this cohort. Comorbidity and functional status were evaluated and incorporated into the modelling which may partly account for frailty within the restrictions of routine data.

### Strengths and weaknesses in relation to other studies

The ACMEplus study examined factors associated with discharge destination at 90 days for 1626 adults aged ≥ 65 years in six European countries [25]. Findings were consistent with our study, in that the majority were discharged home to their previous residence, with predictors of institutional care identified as physical function, living alone, presence of geriatric giants on admission, age and gender [25]. Delirium was not evaluated. Cognitive function was measured using the Katzman score and impairment by this measure did not emerge as a statistically significant predictor of outcomes [25]. The presence of both delirium [26] and dementia [27] has been shown to be associated with the need for institutional care over longitudinal follow-up. Delirium superimposed on dementia has also been shown to be associated with functional dependence and a five-fold risk of care home admission over the year after hospitalisation [28]. These findings are consistent with the associations identified in our study, but the uncertain timing of care home admission after hospitalisation is less directly applicable for patients and their families. The optimal timing of care home admission for individuals with dementia is the subject of considerable interest [29], but one for which research evidence is currently lacking [30]. The pivotal role of caregiver relationships and prevalence of neuropsychiatric symptoms have been examined in international longitudinal community cohorts as important predictors [31], but were not considered in our hospital data. A recent systematic review of the predictors of new institutionalisation after hospitalisation found age, female sex, dementia and functional impairment as significant predictors [14], supporting the view that CSDs are significant. There were no data reported for delirium superimposed on dementia [14]. A 2002 systematic review identified an association between delirium superimposed on dementia and institutional care, but the effect size was not quantified [32]. Therefore, the data from this study add significantly to our understanding of the risk associated with this condition.

### Meaning of the study: possible explanations and implications for clinicians and policymakers

In the face of ageing populations and expected increases in the prevalence of dementia [33], there is an urgent need to improve the care for those with cognitive disorders receive. Furthermore, the recognition that these older people will have complex multimorbidity necessitates a change in the organisation and delivery of acute health care services [34]. This study has taken a pragmatic approach to classifying the cognitive disorders experienced by hospitalised older adults and found evidence of a consistent negative association with being at home 30 days after discharge. While delirium superimposed on dementia was associated with greatest risk, the presence of any CSDs were associated with statistically significant lower likelihood of a positive outcome, indicating a need for further evaluation on the care and support provided to these individuals in the acute hospital setting. These findings highlight the potential risks associated with disease-specific services within acute care, such as dementia units, which may not be accessible by those without a diagnosis such as people with delirium or undiagnosed cognitive impairment who may also benefit from specialised cognition-focused care. Those classified as having unspecified cognitive impairment are an interesting subgroup. Our data do not allow us to establish whether these individuals had undiagnosed dementia, a common finding in hospitalised cohorts where specialist assessment is available [20], or whether they have mild cognitive impairment or subsyndromal delirium. Their reduced likelihood of a positive outcome emphasises the need for early cognitive assessment as a core part of an acute hospital admission.

## Conclusions

There has been increasing recognition of the need for a greater understanding of cognitive spectrum disorders and how these impact on outcomes, particularly for hospitalised older adults [4]. It is recognised that raising awareness through training is not sufficient in addressing the care received and outcomes which result [35]. Research in this area requires an interdisciplinary approach considering mechanisms, detection, care, prevention and the patient/family experience. This requires evaluation of in-hospital processes of care and adverse events which are more common in those with dementia and often under-valued by hospital staff [36, 37]. Recent work has tried to investigate the processes of care and association with excess mortality [38], but no attention has yet been given to how these impact on discharge destination or post-hospital residency and readmission. The elevated risk of those with delirium superimposed on dementia needs further examination to establish how to best support those individuals and their families. We need to understand how to configure and provide healthcare services to enable older people to remain as independent as possible for as long as possible and to ensure transitions of care are managed supportively. Using routine-linked data offers the potential to explore such questions at a population level to facilitate health services research, comparing care models and outcomes and allowing better targeting of specific research projects to explore mechanisms and experiences. This approach should help to develop evidence-based care to support older adults with cognitive spectrum disorders.

## Abbreviations

95%CI: 95% confidence interval
ADL: Activities of daily living
AMU: Acute medical unit
CCI: Charlson Comorbidity Index
CHI: Community Health Index
CSD: Cognitive spectrum disorders
HIC: Health Informatics Centre
NHS: National Health Service
OPRAA: Older Persons Routine Acute Assessment
OR: Odds ratio
PPI: Patient and public involvement
SIMD: Scottish Index of Multiple Deprivation
SMR: Scottish Morbidity Record
